# Blood-based DNA Methylation Biomarkers for Early Detection of Colorectal Cancer

**DOI:** 10.4172/jpb.1000477

**Published:** 2018-06-26

**Authors:** Lixn Dong, Hongmei Ren

**Affiliations:** 1 Mumetel LLC, University Technology Park at IIT, Chicago, IL 60616, USA; 2 Department of Biochemistry & Molecular Biology, Wright State University, 3640 Colonel Glenn Hwy., Dayton, OH 45435-0001, USA

**Keywords:** Colorectal cancer, Blood-based biomarkers, DNA methylation, Early detection

## Abstract

Colorectal cancer (CRC) is a leading cause of cancer-related deaths worldwide. Early detection of CRC can significantly reduce this mortality rate. Unfortunately, recommended screening modalities, including colonoscopy, are hampered by poor patient acceptance, low sensitivity and high cost. Recent studies have demonstrated that colorectal oncogenesis is a multistep event resulting from the accumulation of a variety of genetic and epigenetic changes in colon epithelial cells, which can be reflected by epigenetic alterations in blood. DNA methylation is the most extensively studied dysregulated epigenetic mechanism in CRC. In this review, we focus on current knowledge on DNA methylation as potential blood-based biomarkers for early detection of CRC.

## Introduction

Colorectal cancer (CRC) is a leading cause of death worldwide, accounting for around 754, 000 deaths in 2015 [1], The World Health Organization estimates a substantial increase in the number of newly diagnosed CRC cases worldwide and an 80% rise in deaths from CRC by 2030 [2]. The early detection of CRC significantly improves the prognosis of patients and is a key factor in reducing the mortality of CRC. The cancer can be cured by surgical procedures if it is diagnosed early, specifically before metastasis is established. The 5-year relative survival rate for early-stage CRC is 90%; for advanced stage IV CRC, the rate drops to about 14% [3]. However, only about 4 out of 10 CRC patients are diagnosed at the early stage [4], partially due to poor patient acceptance and/or sensitivity of available screening modalities.

Four types of tests are currently available for CRC detection or screening, including fecal-based occult blood test (FOBT or FIT), tumor marker blood test, combined fecal DNA and FOBT test, and colonoscopy. Colonoscopy screening is currently the standard method for the detection of CRC [5]. However, colonoscopy screening requires bowel preparation and sedation, and is associated with high cost, possible complications and low compliance. The specificity or sensitivity of FOBT is not sufficient [6], and compliance is low due to the inconvenience of sampling and the interference of the test results by many factors [7,8]. Although lab tests such as stool occult blood and recently introduced stool DNA test offer indications for possible CRC, there is a high false positive rate when using those tests. Therefore, robust diagnostic non-invasive biomarkers are urgently needed to detect early stage of CRC.

Both genetic and epigenetic alterations have been found to be involved in the carcinogenesis of CRC [9–11]. The prevailing consensus suggests that epigenetic alterations occur early and more frequently than genetic alterations in CRC [12]. The epigenetic alterations include aberrant DNA methylation, histone modifications and expression of microRNAs (miRNAs) and long non-coding RNAs (IncRNAs) [13]. Post-translational modifications of histones regulate the packaging structure of DNA (called chromatin). Active DNA regions are marked with H3K4me2- or me3 and/or H3, H4 acetylation, while H3K9me3 or H3K27me3 represses genomic regions [14]. Gezer et al. observed reduced plasma levels H3K9me3 and H4K20me3 as potential diagnostic biomarkers for CRC [15]. The miRNA post transcriptionally downregulates gene expression through binding to a complementary site that resides on the 3’-untranslated region of target mRNAs [14]. Many miRNAs associated with CRC diagnosis and prognosis have been identified in patient blood. For example, miR-21 is overexpressed in the plasma or serum of patients with CRC [16], suggesting that it is a promising noninvasive biomarker for the early detection of CRC. Among the epigenetic mechanisms, DNA methylation is the most widely studied and a crucial epigenetic marker in cancer. In this review, we provide an overview of the role of DNA methylation alterations in CRC and discuss the clinical application of these changes as biomarkers for early detection of CRC.

## DNA methylation in CRC

**Figure 1:**
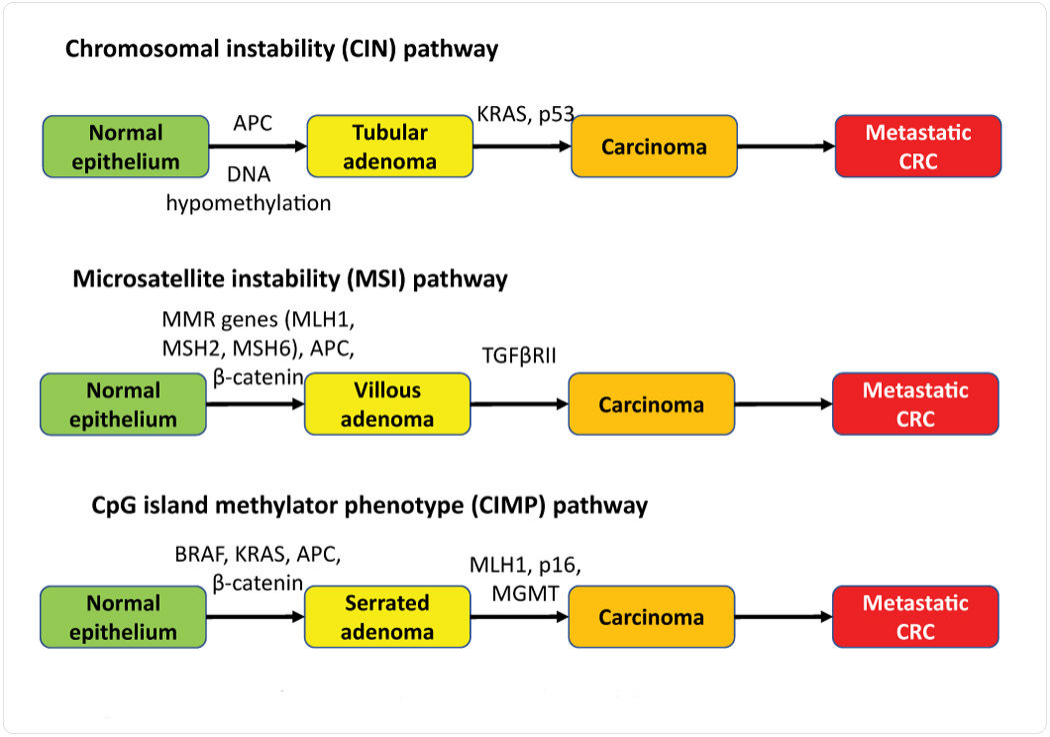
Multiple genetic pathways in colorectal cancer pathogenesis.

CRC results from the accumulation of both genetic and epigenetic changes that transform normal glandular epithelium into invasive adenocarcinoma [17]. Most CRCs develop through two different morphological multistep pathways, including the classical adenoma- carcinoma sequence and the serrated neoplasia pathway [18]. Over the past 25 years, the molecular basis of this process has been progressively clarified. There are at least three distinct molecular pathways in CRC pathogenesis: the chromosomal instability (CIN), microsatellite instability (MSI) and CpG island methylator phenotype (CIMP) pathways (Figure 1) [19]. About 65% of CRC arise through the CIN pathway, which is characterized by widespread imbalances in chromosome number (aneuploidy) and loss of heterozygosity (LOH) [20]. Mutations have been reported in oncogenes and tumor suppressor genes, including adenomatous polyposis coli (APC), β-catenin, K-Ras (KRAS), B-Raf (BRAF), F-box and WD repeat domain containing 7 (FBXW7), transcription factor 7-like 2 (TCF7L2), G protein subunit alpha S (GNAS), chromobox 4 (CBX4), SMAD family member 4 (SMAD4), p53, ADAM metallopeptidase with thrombospondin type 1 motif 18 protein (ADAMTS18), TATA-box binding protein associated factor 1 like (TAF1L), APC membrane recruitment protein 1 (AMER1/ FAM123B), CUB and sushi multiple domains 3 (CSMD3), integrin subunit beta 4 (ITGB4), LDL receptor related protein IB (LRP1B), and spectrin repeat containing nuclear envelope protein 1 (SYNE1) [21]. MSI occurs in around 15% of all CRC tumors and in 90% of CRC occurring in Lynch syndrome patients [22,23]. Mutations in DNA mismatch repair genes (such as MSH2, MLH1, MSH6, and PMS2) result in a failure to repair errors in repetitive sequences, leading to MSI of tumors [24]. Approximately 20% of CRC is associated with CIMP tumors [25–28]. A commonly used panel for defining CIMP is one suggested by Weisenberger et al. which includes neurogenin 1 (NEUROG1), suppressor of cytokine signaling 1 (SOCS1), runt related transcription factor 3 (RUNX3), insulin-like growth factor 2 (IGF2), and calcium voltage-gated channel subunit alphal G (CACNA1G) [29,30]. CIMP-positive tumors exhibit unique clinical, pathological, and molecular features, including a predilection for proximal location in the colon, female gender, poor and mucinous histology, and the presence of frequent KRAS and BRAF mutations [31]. Patterns of mutated genes vary according to the class of CRC. BRAF mutations seem prevalent in MSI [32–34], whereas p53 gene mutations are found in CIN [35]. Despite the differences, these three pathways are not mutually exclusive. A tumor can occasionally exhibit features of multiple pathways. For example, up to 25% of MSI cancers exhibit chromosomal abnormalities [36]; CIMP accounts for most of the MSI- positive CRCs [37]; up to 33% of CIMP-positive tumors exhibit a high degree of chromosomal aberrations and as many as 12% ofCIN-positive tumors exhibit high levels of MSI [38,39]. The most common signaling pathways that carry mutant genes in CRC include the RAS/RAF/ MAPKpathway, the PI3K pathway, the WNT/APC/CTNNB1 pathway and the TGFβl/SMAD pathway [40]. Inactivation of APC leads to upregulation of the Wingless/Wnt pathway, a common mechanism for initiating colorectal adenoma formation [41]. Mutations in KRAS or BRAF aberrantly activate the MAPK signaling pathway, thus inducing proliferation and suppressing apoptosis [42,43].

It is now accepted that DNA methylation alterations are as significant as genetic mutations in driving CRC development. In fact, many more genes are affected by aberrant methylation than by mutations in the average colon cancer genome [12,44,45]. DNA methylation refers to the enzymatic addition of a methyl group to the 5’-position of cytosine by DNA methyltransferases (DNMTs) to produce 5-methylcytosine [46]. The majority of CpG dinucleotides in the human genome are methylated [47,48]. CpG islands indicate regions with at least 200 bp, a GC percentage greater than 50%, and an observed-to-expected CpG ratio > 0.6 [49]. In contrast to CpG dinucleotides, CpG islands typically located in the promoter of proteincoding genes are normally unmethylated in normal healthy cells [50–52]. It has long been established that cancer is characterized by global hypomethylation and hypermethylation at selected CpG islands, which contributes to tumorigenesis by aberrant silencing of tumor suppressor genes [53].

The global DNA hypomethylation is believed to influence CRC development by inducing chromosomal instability and leading to loss of imprinting [54]. The global loss of DNA methylation occurs predominantly within repetitive transposable DNA elements, such as long interspersed nuclear element-1 (LINE-1) and short interspersed transposable element (SINE or Alu elements) sequences [55–61]. DNA hypomethylation can be found in the colon in an age-dependent fashion [62,63] as well as early events in CRC development [64].

## Multiple blood-based DNA methylation biomarkers

Many cells and tissues release some of their constituents to the bloodstream, including fragmented, cell-free DNA (cfDNA) which can also arise from tumor cells, i.e., circulating tumor DNA (ctDNA). Tumor-specific genetic and epigenetic alterations found in cfDNA are likely to represent a mixture of alterations in primary tumor and/or metastatic sites [65]. Cell-free DNA (cfDNA) in the blood circulation of cancer patients (as liquid biopsy) have emerged as key biomarkers for cancer monitoring and treatment decision making [66]. DNA methylation has been used as a diagnostic CRC marker because specific methylation events occurring early in multistep carcinogenesis have been identified and epigenetic gene silencing plays a causative role in CRC development [67–72]. Aberrant DNA methylation occurs in the blood of adenoma patients, making DNA methylation biomarkers feasible to detect CRC early [73,74]. Blood-based DNA methylation is mainly derived from cell-free nucleic acid released from circulating cells in serum or plasma or DNA extracted from peripheral blood leukocytes or whole blood cells.

## Septin-9 (SEPT9)

SEPT9 is one of the most extensively studied genes as a blood- based biomarker for CRC patients [75–80]. It belongs to the gene family that encodes a group of GTP-binding and filament-forming proteins involved in cytoskeletal formation and cell cycle control [81]. It has promoter hypermethylation reaching sensitivities ranging from 51% to 90.0%, and the specificity from 73% to 96% in serum or plasma samples of CRC patients [75–80]. However, the sensitivity of the methylated SEPT9 assay in detecting advanced adenomas is low (9.6%) [79], suggesting that this gene alone might be of limited value in detecting precancerous lesions.

## Human MutL homolog 1 (MLH1)

As described earlier, MLH1 is linked to MSI in CRC [22]. Grady et al. found aberrant hypermethylation of the MLH1 promoter in the sera of 9 out of 19 (47%) cases of CRC [82]. Leung et al. monitored promoter hypermethylation in three genes, APC, MLH1, and helicase-like transcription factor (HLTF), and found at least one of the three genes with methylated promoter DNA in the sera of 28 out of 49 CRC patients, which gave a sensitivity of 57% and specificity of 90% [83].

## APC

APC gene promoter hypermethylation has been described to explain the sustained activation of the Wnt signaling pathway [84]. PARK et al. [85] examined the methylation status of the APC gene along with other 4 genes including mothers against decapentaplegic homolog 4 (SMAD4), fragile histidine triad protein (FHIT), death-associated protein kinase 1 (DAPK1), and E-cadherin in the peripheral blood plasma of 60 CRC patients, 40 patients with adenomatous and 60 healthy controls using methylation-specific PCR single-strand conformation polymorphism (MSP-SSCP) analysis. The APC marker displayed a sensitivity of 57% for the detection of CRC at a specificity of 86%, and a sensitivity of 57% and specificity of 89% in stage I of CRC.

## Cyclin dependent kinase inhibitor 2A (CDKN2A)/p16

CDKN2A is an inhibitor of cyclin-dependent kinase 4 (CDK4) and CDK6, and it functions as a tumor suppressor [86]. Furthermore, it is among the panel of surrogate markers used to evaluate CIMP phenotype [87]. The studies of Zou et al. [88], Nakayama et al. [89] and Lecomte et al. [90] examined the aberrant promoter hypermethylation of CDKN2A (p16) in serum of CRC patients and yielded 70% (23 out of 34 patients), 69% (31 out of 45 patients) and 68% (31 out of 45 patients) sensitivity, respectively. Nakayama et al. [91] further analyzed CDKN2A/p16 hypermethylation as a marker for CRC recurrence; 8 out of 21 CRC detected p16 hypermethylation in preoperative serum samples and 13 out of 21 CRC detected p16 hypermethylation in primary tumor biopsies suggesting its potential role in recurrence of CRC.

## LINE-1

In addition to hypermethylated genes, DNA hypomethylation status of genes is associated with prognosis of CRC patients. LINE-1 repeat elements were progressively hypomethylated in the normal-adenoma- cancer sequence [92]. Nagai et al. [93] examined 114 plasma samples of CRC patients, and quantified LINE-1 hypomethylation status in plasma cfDNA by absolute quantitative analysis of methylated alleles (AQAMA) real-time PCR. Detection of early stage I/II CRC through cfDNA LINE-1 hypomethylation index (LHI) was accomplished with 63.2% sensitivity and 90.0% specificity, suggesting the potential utility of cfDNA LHI as a blood biomarker for early CRC detection [93]. The efficacy of this assay has been validated in several previous studies [60,61,94,95].

DNA methylation is involved in the process of CRC initiation, progression and metastasis. DNA methylation biomarkers discriminate among clinical stages and predict disease progression. For example, in early stages of the serrated pathway, mutation of BRAF elevates the expression of tumor suppressor genes p16 and insulin-like growth factor-binding protein 7 (IGFBP7) holding the microvesicular hyperplastic polyp (MVHP) to a small and nonprogressive lesion [96]. Aberrant CpG island methylation of the promoter region of p16 and IGFBP7 bypasses this dormant state and drives MVHPs further to sessile serrated adenomas (SSAs) [97]. In blood samples, hypermethylated ALX4 [80,98], NEUROG1 [99], APC [100], 6-O-Methylguanine-DNA Methyltransferase (MGMT) [101], MLH1 [82], HLTF [102], Ras association domain family member 2 (RASSF2A) [101], Syndecan 2 (SDC2) [103], SEPT9, Preprotachykinin-1 (TAC1) [104] and WIF1 [101] were detected in early stage CRC; hypermethylated HPP1, HLTF, secreted Frizzled Related Protein 2 (SFRP2) [105], VIM [106], tissue factor pathway inhibitor 2 (TFPI2) [107,108] and p16 [89] were found positively correlated with distant tumor metastasis; hypermethylated ALX4, fibrillin-2 (FBN2), HPP1 (Alias TMEFF2) [102], HLTF [83], p16 [91], TMEFF1 [102] and VIM [99] were associated with poor prognosis; and hypermethylated HLTF [109], HPP1 [109], runt- related transcription factor 3 (RUNX3) [110], p16 [91] and TFPI2 [107,108] were associated with CRC recurrence. It is conceivable that a robust biomarker panel of methylated genes will be developed into a clinically accurate CRC screening method in the future, and the development of blood-based biomarkers should improve patient compliance and the detection of CRC at early stage.

Until now, only one blood-based assay that detects methylated SEPT9 was approved by the U.S. Food and Drug Administration for CRC screening under the name Epi proColon® (Epigenomics, Berlin, Germany). In a screening-like cohort study, the assay was compared to the reference standard FIT test (100 ng/mL cutoff) in 97 CRC cases (stage I-IV) and 193 non-CRC controls [111]. Epi proColon 2.0 had a sensitivity of 72.2% with a specificity of 80.8%. Conversely, the FIT test had a sensitivity of 68% at a specificity of 97.4% [111]. SEPT9 combined with ALX4 and HPP1 was tested in plasma from 182 CRC cases (stage I-III) and 170 healthy controls and yielded a sensitivity of 80.7% at a specificity of 90% [112]. Various DNA methylation biomarkers reported in clinical studies have been listed in Table 1. Larger clinical trials are needed to further validate these gene biomarkers.

Using ctDNA for early cancer diagnosis is challenging due to the low amount of tumor DNA released in the circulation. The recent development of new technologies such as droplet-based digital PCR (ddPCR) or next generation sequencing (NGS) has greatly improved the sensitivity and specificity for the detection of tumor-specific alterations [113]. The bisulfite sequencing provides single base resolution for broad profiling of DNA methylation, whereas ddPCR allows absolute quantification of target DNA methylation and may be suited for clinical decision-making.

## Discussion and Conclusion

CRC continues to be a significant public health burden and the 5-year prognosis for metastatic CRC is still less than 15% [3]. Aberrant methylation of specific genes measured in blood samples could be used as a CRC biomarker and provide prognostic information. CRC is a heterogeneous disease and DNA methylation biomarkers based on single gene have limited sensitivity and specificity. Further studies are therefore needed to perform a genome-wide search to produce a panel of sensitive and specific DNA methylation markers for the early detection of CRC.

## Figures and Tables

**Table 1:** Overview of blood-based biomarker in clinical studies of CRC.

Gene	Name	Pathway	Sample size (CRC/Control)	Sensitivity	Specificity	Reference
SEPT9	Septin9	Cell cycle control, ERK signaling and bacterial invasion of epithelial cells	53/1457	48.2	91.5	Church [79]
			1031	76.6	95.9	Wu [114]
			252/102	48	90	Grutzmann [115]
			85/324	38.7	81	Song [116]
			44/1500	68	-	Potter [117]
			135/185	69	86	Lofton-Day [77]
IKZF1/BCAT1	Ikaros family zinc finger protein 1/ Branched-chain amino acid transaminase 1	Viral mRNA translation and metabolism	129/1976	66	95	Pedersen [118]
			66/1315	62	92	Symonds [119]
WIF-1/NPY	WNT inhibitory factor 1/Neuropeptide Y	Notch and Wnt signaling	243/276	86.5	92.1	Lee [101]
ALX4	Aristaless-like homeobox 4	DNA binding transcription factor activity and protein heterodimerization activity	30/30	83.3	70	Ebert [120]
			135/185	69	86	Lofton-Day [77]
			193/102	90.7	72.5	Rasmussen [121]
			182/170	48	87	He [112]
NEUROG1	Neurogenin 1	Neural crest differentiation and signaling pathways regulating pluripotency of stem cells	252/93	61	91	Herbst [99]
VIM	Vimentin	Wnt signaling pathway	81/110	59	93	Li [122]
			239/25	32.6	-	Shirahata [123]
			193/102	90.7	72.5	Rasmussen [121]
NGFR	Nerve growth factor receptor	Apoptotic pathways in synovial fibroblasts and p75 (NTR) - mediated signaling	133/179	51	84	Lofton-Day [77]
HPP1 (TMEFF2)	Hyperpigmentation, progressive, 1	Validated targets of C-MYC transcriptional repression	133/179	65	65	Lofton-Day [77]
			95/32	20	93.7	Herbst [99]
			182/170	81	90	He [112]
			38/20	18	100	Wallner [124]
MLH1	mutL homolog 1	DNA mismatch repair pathway	38/20	21	100	Wallner [124]
			49/41	43	98	Leung [83]
CDKN2A (p16)	Cyclin dependent kinase inhibitor 2A	Cell cycle regulatory pathway	44/50	68	-	Nakayama [125]
